# Colliding Pandemics and CoViD-19

**DOI:** 10.6026/97320630019251

**Published:** 2023-03-31

**Authors:** Lily Fotovat, Francesco Chiappelli

**Affiliations:** 1 Dental Group of Sherman Oaks, CA 91403, USA (www.oliviacajulisdds.com); 2 Center for the Health Sciences, UCLA, Los Angeles, CA; Dental Group of Sherman Oaks, CA 91403

**Keywords:** World Health Organization (WHO), Center for Disease Control (CDC), Systemic Acute Respiratory Syndrome Corona virus-2 (SARS-CoV2), Spike (S) protein, Corona Virus Disease 2019 (CoViD-19), Respiratory Syncytial Virus (RSV), fusion-associated small transmembrane proteins (FAST), monkeypox virus (MPXV), Influenza A, B & C Virus (IAV, IBV, ICV), viral surface proteins: hemagglutinin (H) and neuraminidase (N) influenza viral surface proteins, Centers for Disease Control (CDC), oral manifestations, viral recombination, viral inference

## Abstract

Cases of the respiratory syncytial virus (RSV), monkeypox virus (MPXV), and avian influenza A Virus (IAV) have increased during our current prolonged Corona Virus Disease 2019 (CoViD-19) pandemic. The rise of these viral infectious diseases may be associated or even inter-dependent with acute, latent or recurrent infection with Systemic Acute Respiratory Syndrome Corona virus-2 (SARS-CoV2). The nonsensical neologism 'tripledemic' was tentatively introduced to describe the confluent nature of these trends (epidemic comes from two Greek words: *epi*=on, about, *demos*=people; pandemic is also derived from Ancient Greek: *pan*=all, *demos*=people; but 'tripledemic' would derive from Latin *triplus*=three, Greek *demos*=people, and would at best signify 'three countries, three peoples', but certainly not the current threat of confluence of three, or perhaps more pandemics). Emerging evidence suggests that monkey pox and CoViD-19, among several other viral diseases, produce significant observable manifestations in the oral cavity. From a clinical standpoint, dentists and dental personnel may be among the first health professionals to encounter and diagnose clinical signs of converging infections. From the immune surveillance viewpoint, viral recombination and viral interference among these infectious diseases must be examined to determine the potential threat of these colliding pandemics.

## Background:

## Respiratory Syncytial Virus (RSV):

Human RSV is a non-segmented negative sense single strand-RNA *Orthopneumovirus* (i.e., targeting the upper respiratory tract) of the genus *Orthopneumovirus*, family *Pneumoviridae* and order *Mononegavirales*. It is characterized by having membrane fusion-associated small transmembrane proteins (FAST) on its surface, which lead neighboring cell membranes to merge into large multi-nucleated syncytia. FAST proteins are Class IV viral surface ligand proteins, and akin to Class I viral fusion proteins, such as the SARS-CoV2 Spike (S) protein, and influenza A virus (IAV) hemagglutinin (H) surface protein.

RSV typically affects the lungs and breathing passages in children and adults. In the younger pediatric population, RSV is the major cause of respiratory tract illness with a wide range of clinical symptoms, including upper respiratory tract infections, severe lower respiratory tract infections, and exacerbation of underlying disease of pandemic proportion with almost 33.8 million cases worldwide in children under five years of age [1,2, 3]. Seasonal convergence of influenza and RSV *epi*demics contributes to high morbidity and mortality rates among children [4]. Although life-threatening infections most commonly occur during the first few years of life, RSV infections can affect older children and adults [2].

The oral cavity, nasal passages and airways are anatomically large surfaces that interact with the surrounding environment, and serve as reservoirs for bacteria, and viruses. Case in point, RSV can increase the virulence of potential pathogens in the nasopharynx, a main reservoir of bacteria, promoting bacterial diseases to spread to the lungs [5]. Elevated RSV viral loads in the nasopharynx enable faster transmission between individuals because of active viral shedding via the eyes and nose [6]. In severe RSV infection, changes in the intestinal microbiota indirectly impact the of mucosa distal to the gastrointestinal tract [2].

During the CoViD-19 pandemic, the pattern of spread of RSV has changed dramatically. Lockdowns in 2020 in the northern hemisphere appear to have decreased transmission, delaying RSV peaks during 2022/21 [7]. The major interventions at a global level were stay-at-home orders, social distancing, and basic public health measures, such as social distancing, hand hygiene and mandatory face masks which have contributed to reducing the risk of RSV air droplets and fomites transmission [8].

Public health measures may have reduced usual exposure to pathogens, and limited the ability of children and adults to mount immune surveillance and resistance to certain viruses, including RSV and IAV. Case in point, RSV-positive acute respiratory infections have reemerged later in the CoViD-19 pandemic and during the non-RSV season of 2021-2 [9]. A 3% increase of patients that were hospitalized with CoViD-19, ranging from infants to adults, were also co-infected with RSV, and possibly IAV) [10]. It has been proposed that the increase in an immunologically susceptible population, with infants born from mothers immunologically susceptible to RSV, may lead to greater risk of RSV epidemics in the future [8].

## Monkeypox Virus (MPXV):

Monkeypox Virus (MPXV) is a double-stranded DNA virus of the pox family (*poxviridae*) [11]. It belongs to the orthopoxvirus genus, like smallpox (i.e., variola), cowpox, and vaccinia viruses. MPXV is a rarely fatal zoonotic disease, endemic in Western and Central Africa (e.g., central Congo), and only recently in Asia, Western Europe, Australia and the US.

Human zoonotic infection with MPXV usually occur via infected animals, mainly rodents: squirrels, Gambian pouched rats, and dormice [12]. MPXV can also be transmitted through direct anthropogenic human-to-human contact via respiratory droplets, direct contact with infectious sores, scabs, bodily fluids, and fomites in shared bedding/clothing. Sexual transmission of MPXV occurs via skin lesions; but MPXV is not a sexually transmitted disease [12].

The incubation period is generally between 6 and 13 days. Prodromal symptoms of MPXV start with the emergence of rashes and sores manifest as minor to moderate flu-like symptoms that can last up to two to four weeks: body aches (i.e., muscle and joint pains), chills, fatigue, fever, headaches, and swollen lymph nodes. Patients are considered infectious only when distinct rashes develop, including sores and lesions resembling pimples or spots [13].

The 2022-3 MPXV pandemic outbreak involved multiple countries and continents in endemic and non-endemic regions [14]. Most cases have been traced to sexual transmission, especially men who have sex with men ages 20 to 50 [12]. Current data suggest that adult gay and bisexual males, regardless of race/ethnicity, age, gender identity, or other characteristics, make up most cases in the current outbreak [12].

Current pandemic manifestations have revealed characteristic of oral and oropharyngeal lesions that appear concurrently with the first skin lesions and rashes, with accompanying fever and swollen lymph nodes [15]. MPXV-positive patients may manifest vesicular or pustular lesions in the oral cavity, often with oral blisters of a spherical shape and a crimson border after breaking off the roofs of the vesicle or pustule. The sores are initially painful and later itchy [16]. The Centers for Disease Control (CDC) has confirmed that 70% of MPXV patients had some form of mouth or tongue lesions [15].

Taken together, these data suggest that saliva can harbor the MPXV, and transmission can occur through oral-skin and/oral-anogenital contact [15]. These observations also indicate that dentists and dental personnel may be not only the first health professionals to encounter and diagnose MPXV manifestations in their patients [15], but also the health professionals most at risk of inadvertent infection.

## Influenza A, B and C Virus (IAV, IBV, ICV):

Influenza is caused by one of the seven members of the influenza virus family (i.e., *genera*: α-influenzavirus, β-influenzavirus, γ-influenzavirus, δ-influenzavirus, Isavirus, Thogotovirus, and Quaranjavirus). The four principal *genera*, with respect to human disease, each containing a single species, are referred to as or type Influenza A Influenza virus A, IVA) and C, which are zoonotic and infect a variety of species, including birds and humans, anthropogenic influenza B infectious primarily to humans, and influenza D that infects cattle and pigs. Influenza viruses are negative sense single-strand RNA viruses with a segmented genome. Influenza A and influenza B viruses contain eight RNA segments that encode for RNA polymerase subunits and viral glycoproteins, including (H), which facilitates viral entry, and neuraminidase (N), which facilitates viral release and shedding. The viral genome also encodes for the viral NP nucleoprotein, the M1 matrix protein and the M2 membrane protein, the NS1 nonstructural protein, and the NEP nuclear export protein [17,18, 19].

IAV sub-groups are classified based on the 18 serotypes of the hemagglutinin (H) protein, and the 11 serotypes of the neuraminidase (N) protein (e.g., H1N1, causative agent for the 1918-20 Spanish flu and 2009-10 Swine flu pandemics, H3N2, causative agent if the 1968-9 Hong Kong flu pandemic, H5N1, Asian flu so-called responsible for the 2005-7 avian disease, and presently emerging imminent pandemic threat).m Anthropogenic infection typically happens via droplets, when the virus gets into a person's eyes, nose or mouth, or is inhaled [18]. Symptoms of infection typically include acute mild to severe, and potentially even lethal respiratory disease characterized by fever, sore throat, runny nose, cough, headache, and fatigue [17].

Since the beginning of 2023, poultry farms have seen H5N1 outbreaks, which are a particularly significant threat because zoonotic infection (i.e., animal-to-human) is common placing farm workers at heightened risk, and because H5N1 (e.g., Asian-lineage High Pathogenic A Influenza virus [HPAI] H5N1) can kill over half the humans it infects. The North American low pathogenic avian H5N1 - LPAI H5N1 - causes minor sickness in birds, and is not known to infect humans. H5N1 HPAI, which infects domesticated birds (i.e., chicken) are a potential threat to food security [20], whereas LPAI H5N1 infects mostly wild avian populations.

H5N1 vaccines exist, which can help protect certain endangered wild birds (e.g., bald eagle) [20]. In addition, research programs have been geared toward the development and testing of pre-pandemic vaccines, if and when such need should arise. The recent death of a girl in Cambodia, and her father's positive test for H5N1 have ignited concerns for an H5N1 epidemic in Asia, and a possible global pandemic. While the risk of such a development is still currently low, it is certainly concerning in light of recent experience with the ongoing CoViD-19 pandemic [20]. Moreover, Bolivian government authorities have announced that ten to a dozen people were possibly infected with H5N1 as of early March 2023 - so far localized to the Province of Nor Chichas in the Department of Potosí (https://elpotosi.net/local/20230306_gripe-aviar-aisla-a-comunidad-que-tuvo-contacto-con-aves.html).

## Conclusion:

The current CoViD-19 pandemic results from infection with the highly transmittable member of the Corona family, the positive-sense single-stranded RNA SARS-CoV2 virus. Corona viruses are among the largest non-segmented RNA viruses, with a genome endowed with the 5'methylated cap and 3' polyadenylated tail critical for translational events.

SARS-CoV2 is an airborne virus that spreads primarily through respiratory droplets from spit produced when an infected person coughs, sneezes, or talks [21,22]. Saliva has been used as an effective form of CoViD-19 diagnosis; therefore, the human oral cavity can potentially serve as a reservoir of the SARS-CoV2 virus. Researchers suggest that CoViD-19 can use severe oral mucosa lesions and is likely connected to poor periodontal health [23]. Cases of CoViD-19 patients have been reported where severe oral mucosa lesions manifest, including the detachment of oral epithelium with inflammation and fibrosis, and putatively periodontal disease from CoViD-19 [23]. However, a SARS-CoV2 infection is rapid and difficult to identify if the deeper cells, like the fibroblasts, are infected directly [23]. To better understand this relationship, dentists and dental personnel should be informed whether or not their patient had CoViD-19 and was simultaneously diagnosed with periodontal disease. It is possible and even likely that oral manifestations of CoViD-19 will be more evident in patients with Long Covid (i.e., PACS [24]).

It is not uncommon that multiple respiratory viruses, such as Corona viruses (i.e., SARS-CoV2), influenza viruses (i.e., IAV, H5N1) or RSV can concurrently or sequentially infect the respiratory tract [22]. These types of co-infections may lead to virus-virus interactions [22] or even recombination of the viral genomes with concomitant exchange of genetic material between at least two separate viral genomes. Viral recombination of this sort occurs in both positive-strand RNA viruses (e.g., SARS-CoV-2) and negative-strand RNA viruses (e.g., RSV, IAV), when two or more such viruses co-infect a single cell and exchange segments. The resulting outcomes can be viable viral hybrid progeny that combine the properties (i.e., infectivity, transmissibility, shedding, vaccine escape) of the original viruses [25]. It is likely and even probable that recombinant RNA viruses with SARS-CoV2 and/or H5N1 will be presently be identified. Co-infection of SARS-CoV2 and MPXV has been reported, with the characteristic vesicular and ulcerative lesions in the genital lesions, observed in Monkeypox, but generally not in CoViD-19 [26].

It is also not common that one viral infection may protect the organism against another: a viral interference process mediated at the molecular level of by γ-interferon-modulated immune surveillance [27]. Several factors may contribute to determining viral interference. It is possible that the process of interference of one virus in a co-infection paradigm may counteract, or perhaps even aid viral interference of the other co-infecting virus. Alternatively, viral camouflage may result an escape from immune surveillance [28]. It is likely that in situations of co-infection by two or more viruses, one viral particle may aid, or favor the escape of another.

In conclusion, when viral epidemics or pandemics collide, as is the case presently with SARS-CoV2, and RSV, MPXV, IAV, and perhaps pathogens yet to be determined, several complex, important, and intertwined events ensue, which include the individual pathology caused by one predominant virus, the combination of pathologies of the co-infecting viruses, and new and putative pathologies resulting from novel hybrid recombinant viral species. It is critical and timely that concerted efforts be channeled toward a full elucidation of the possible and even likely mixed and nested outcomes of pandemics presently emerging and colliding with CoViD-19.
